# Calculated parenteral initial treatment of bacterial infections: Intra-abdominal infections

**DOI:** 10.3205/id000057

**Published:** 2020-03-26

**Authors:** Christian Eckmann, Rainer Isenmann, Peter Kujath, Annette Pross, Arne C. Rodloff, Franz-Josef Schmitz

**Affiliations:** 1 Klinik für Allgemein-, Viszeral- und Thoraxchirurgie, Klinikum Hannoversch-Münden, Germany; 2 Allgemein- und Visceralchirurgie, St. Anna-Virngrund-Klinik Ellwangen, Germany; 3 Chirurgische Klinik, Medizinische Universität Lübeck, Germany; 4 Klinik und Poliklinik für Chirurgie, Universitätsklinikum Regensburg, Germany; 5 Institut für Medizinische Mikrobiologie und Infektionsepidemiologie, Universitätsklinikum Leipzig, Germany; 6 Institut für Laboratoriumsmedizin, Mikrobiologie, Hygiene, Umweltmedizin und Transfusionsmedizin Johannes Wesling Klinikum Minden, Germany

## Abstract

This is the seventh chapter of the guideline “Calculated initial parenteral treatment of bacterial infections in adults – update 2018” in the 2^nd^ updated version. The German guideline by the Paul-Ehrlich-Gesellschaft für Chemotherapie e.V. (PEG) has been translated to address an international audience.

The chapter deals with the empirical and targeted antimicrobial therapy of complicated intra-abdominal infections. It includes recommendations for antibacterial and antifungal treatment.

## Zusammenfassung

Dies ist das siebte Kapitel der von der Paul-Ehrlich-Gesellschaft für Chemotherapie e.V. (PEG) herausgegebenen S2k Leitlinie „Kalkulierte parenterale Initialtherapie bakterieller Erkrankungen bei Erwachsenen – Update 2018“ in der 2. aktualisierten Fassung.

Es behandelt die empirische und gezielte antimikrobielle Therapie komplizierter intraabdominaler Infektionen und enthält Empfehlungen für die antibakterielle und antimykotische Behandlung.

## Indications for antimicrobial treatment

Intra-abdominal infections (IAI) are common. Diagnosis of acute peritonitis is very common. The Institute for the Hospital Remuneration System (InEK) recorded 30,000 cases of peritonitis in the area of visceral surgery (MDC 6–7) in 2014. National and international databases show that about 30% of all cases of severe sepsis or septic shock are due to IAI [1], [2], [3]. Nearly 90% of all intra-abdominal infections primarily require surgical infectious source rehabilitation (e.g. hyperalimentation of a gastric perforation). Nevertheless the value of antibiotic treatment versus placebo is confirmed even in this disease group [4]. An initially inadequate antibiotic treatment of IAI substantially worsens the prognosis of the affected patients and leads to considerable economic damage [5], [6], [7], [8]. 

Recommendations for antibiotic treatment in intra-abdominal infections are derived from a variety of prospective randomized and controlled trials. Since the goal of almost all studies is to demonstrate therapeutic equivalence, the current results are insufficient and don’t allow identification of a preferred substance or a substance regimen [4]. It should be added that in all randomized studies the inclusion and exclusion criteria were chosen so that patients with a less severe IAI (APACHE II score around 6) were recruited. This complicates the assessment of the effectiveness of the specified substances in life-threatening peritonitis. When selecting an appropriate antibiotic, the individual patient (e.g. immunosuppression, prior treatment), expected pathogen spectrum, local pathogen and resistance statistics, a simple mode of application, low toxicity of the substances and costs should be considered in the decision-making process.

By definition, complicated IAIs occur when the infection moves beyond the affected organ and causes an abscess or peritonitis (local or diffuse) [6]. However, clinically this differentiation is not clear. For example, phlegmonous appendicitis with low environmental response (lethality below 0.5%) constitutes a complicated IAI, whereas severe *Clostridium difficile*-induced colitis (lethality up to 40% in case of ribotype 027) represents an uncomplicated IAI as per the above definition. Clinically speaking, three different forms of peritonitis can be differentiated, which are causally pathogenetic, substantially different in terms of the spectrum of pathogens and surgical and antimicrobial treatment [9].

## Peritonitis

### Primary peritonitis

Primary (spontaneous bacterial) peritonitis (SBP) affects only about 1% of all peritonitis cases. The juvenile form is a hematogenous infection caused by streptococci, pneumococci or, more rarely, *Haemophilus influenzae*. In adults, predominantly patients with ascites through alcoholic liver cirrhosis (ca. 70%) are affected or patients with a reduced immune status from another cause (ca. 30%) [10], [11]. Mostly it is a mono-infection. In realistic studies, it is only possible to detect pathogens in about 35% of cases, with *Escherichia coli*, *Klebsiella* spp., Staphylococci, enterococci or streptococci, and occasionally pathogenic gastroenteritis pathogens such as *Aeromonas* spp. or *Salmonella* spp. being detected [10]. Primary peritonitis, which can occur as part of tuberculosis, is spread hematogenously.

Randomized studies on the treatment of SBP are rare. Most of them are retrospective studies. Substances used were ceftriaxone, cefotaxime, ceftazidime, ampicillin/sulbactam, ampicillin + tobramycin and amoxycillin/clavulanic acid [12], [13], [14] (Table 1 (Tab. 1)). Using these alongside administration of albumin, clinical cure rates of about 80% were achieved [15]. Regarding treatment of primary peritonitis caused by resistant pathogens, see also Table 1 (Tab. 1).

### Peritonitis under CAPD

CAPD peritonitis is usually caused by contamination of the tubing or catheter system. The most common pathogens are coagulase-negative staphylococci and *Staphylococcus aureus*. *Escherichia coli*, enterococci, streptococci, *Pseudomonas aeruginosa*, anaerobes, Enterobacter spp., or *Candida* species are detected less commonly [16]. Uncomplicated cases can be treated locally by adding antimicrobial substances to the dialysis fluid. In addition to intraperitoneal treatment, parenteral treatment also becomes necessary in the rarer severe forms. The peculiarities of antibiotic dosing in cases of renal insufficiency must be taken into account. 

Cefotaxime, cefuroxime or ceftriaxone (in monotherapy or in combination with ciprofloxacin) is recommended for calculated treatment [17]. Once the results of microbiological diagnostics have been obtained, treatment should continue in a targeted fashion. If MRSA, MRSE and enterococci (including VRE) are detected, the antibiotics in Table 2 (Tab. 2) are available. If the infection is not under control after one week of antimicrobial treatment, the peritoneal dialysis catheter should be removed [18].

### Secondary peritonitis

Secondary peritonitis, with perforation of the gastrointestinal tract, is by far the most common IAI, at around 80–90%. By definition, surgical source control must be carried out (infectious source rehabilitation, for example appendectomy for perforated appendicitis) or interventional treatment (for example CT-controlled drainage of an abscess). In terms of a three-pillar model, diffuse peritonitis requires surgical, antimicrobial and intensive care treatment [18]. Increasingly, primary infectious source rehabilitation is followed by definitive closure of the abdomen and clinical progress monitoring of the patient [19]. In secondary peritonitis, a distinction can be made between a community-acquired (ca. 60%) and a post-operative form (ca. 40%).

### Community-acquired secondary peritonitis

In community-acquired secondary peritonitis there is always a mixed infection. The pathogen spectrum derives from the flora of the gastrointestinal tract and is dependent on the pathogenesis and the location of the perforation or leakage. Key pathogens are *Escherichia coli*, *Bacteroides fragilis*, Enterococci and *Candida* spp. Resistant species need only be considered in patients treated with antibiotics on an out-patient basis and other specific risk factors (see Table 3 (Tab. 3)). The present recommendations take into account the duration of the illness and the pathogen spectrum depending on the cause of the disease [18]. For antibiotic treatment of localized acute peritonitis, cefuroxime, cefotaxime, ceftriaxone or ciprofloxacin, in combination with metronidazole, as well as ampicillin/sulbactam or amoxicillin/clavulanic acid can be used. Piperacillin/tazobactam and ertapenem, which have also been approved and tested in this indication, should be used in cases of more severe IAI (Table 4 (Tab. 4)).

For the treatment of diffuse peritonitis, which persists for more than 2–4 hours, substances or combinations with a broad action spectrum should be used. Piperacillin/tazobactam, moxifloxacin, tigecycline or ertapenem can be used for calculated treatment. Alternatively, combinations of metronidazole with ceftriaxone or cefepime can be used. Considering enterococci in substance selection is recommended only in the exceptional case of known colonization [20], [21], [22], [23]. 

Addition of aminoglycosides did result in improved effectiveness in meta-analyzes and is therefore no longer considered the treatment of choice [24]. Variable kinetic parameters as well as ototoxicity and nephrotoxicity also require regular serum-level control.

### Post-operative, post-traumatic and post-interventional peritonitis

Post-operative peritonitis is a nosocomially acquired secondary form of peritonitis and is defined as an infectious abdominal complication following surgery (for example anastomotic leakage following anterior rectal resection). In post-operative peritonitis, in contrast to tertiary peritonitis, a surgical or interventional (such as an endo-VAC insert into an insufficiency cavity) is a condition in need of treatment [9]. Most patients will already have had antimicrobial treatment at the time of illness. Post-operative peritonitis is thus characterized by a selective pathogen spectrum with enterococci (including VRE), Gram-negative pathogens (“extended spectrum” beta-lactamase [ESBL]) and fungi. *Pseudomonas* spp. and carbapenemase producers are rarely detected.

Imipenem/cilastatin, meropenem, ertapenem, tigecycline and fosfomycin can be used as antibiotics with a broad action spectrum [20], [21], [22], [23], the latter not being used in monotherapy because of the rapid development of resistance. Ceftolozane/tazobactam has recently become available as a new ESBL-effective drug. In the approval study for IAI, which used ceftolozane/tazobactam in combination with metronidazole, this group of patients in particular was treated very successfully under controlled clinical conditions [25], [26]. Another treatment option in this indication range is the recently approved ceftazidime/avibactam in combination with metronidazole. The possibility of fungal infections in empirical anti-infective treatment must also be considered (see Table 5 (Tab. 5)).

### Tertiary peritonits

In tertiary peritonitis (such as post-operative peritonitis, a nosocomial form of peritonitis), infection of the abdominal cavity persists without a focus that can be remedied surgically, after previously completed infectious source rehabilitation of secondary peritonitis [9], [20], [21]. The transitions from secondary to tertiary peritonitis can be fluid. In diagnostically unclear situations, relaparotomy is the only way of proving there is no need for a surgical intervention [21]. Most are low-virulence pathogens, which lead to a sustained infection because of the sustained immunosuppression of the affected patient. This form of nosocomial peritonitis has a similar pathogen spectrum to secondary post-operative peritonitis due to prior antimicrobial treatment. There are often enterococci including VRE, staphylococci incl. MRSA, Enterobacteriaceae incl. ESBL producers and anaerobes. In tertiary peritonitis, *Pseudomonas* spp. and *Candida* spp. are detected more frequently [9], [20], [21], [22]. For antibiotic treatment, tigecycline (possibly in combination with a *Pseudomonas*-active substance) as well as imipenem/cilastatin, meropenem, ceftolozane/tazobactam with metronidazole or ceftazidime/avibactam with metronidazole (if necessary in combination with linezolid) are available [20], [21], [22], [27].

## Necrotizing pancreatitis with infected necroses

About 80% of all deaths from acute pancreatitis are caused by septic complications. The translocation of pathogens from the colon into the peripancreatic tissue is the most common cause of secondarily infected pancreatic necrosis [28], [29], [30]. Infected pancreatic necroses can be suspected if fever, leukocytosis, CRP serum elevation and unexpected clinical deterioration occur. Detection of gas inclusions within necrotic pancreatic tissue in abdominal CT is considered to be evidence of infected necroses [31]. Interventions in cases of infected pancreatic necrosis includes conservative measures (endoscopically guided transgastric drainage, CT-guided drainage) as well as surgical intervention. Currently, it is assumed that the optimal time for surgical treatment (open or minimally invasive) is after more than three weeks [32]. Meta-analyzes already concluded in 2004 and 2006 that a general administration of antibiotics has no significant positive effect on the course of necrotizing pancreatitis and instead leads to a selection of resistant pathogens and *Candida* spp. [33], [34], [35], [36], [37], [38]. The recent guideline of the American College of Gastroenterology recommends that in principle no antibiotic treatment should be carried out [39]. 

A safe indication for antibiotic treatment is proven infected necrosis, infected pseudocysts, abscess formation, cholangitis, and other extra-pancreatic infections. The most important pathogens in infected pancreatic necroses are Enterobacteriaceae, enterococci, staphylococci, anaerobes and *Candida* spp. When selecting appropriate antibiotics, pancreatic mobility of the drugs should also be considered (Table 5 (Tab. 5)). Studies with reliable data for good penetration into the pancreatic tissue exist for fluoroquinolones (ciprofloxacin, moxifloxacin), carbapenems (imipenem/cilastatin, meropenem, ertapenem), tigecycline and piperacillin/tazobactam. Inadequate tissue penetration has been demonstrated for aminoglycosides [18], [24]. All the above-mentioned substances can in rare cases cause pancreatitis.

## Invasive intra-abdominal mycoses

Most intra-abdominal invasive mycoses (IIM) are triggered by *Candida* spp. In total, up to 18% of all severe sepsis cases in Germany were found to be due to *Candida* spp. [3]. One-off detection in surgically-obtained material in cases of community-acquired secondary peritonitis (for example perforated gastric ulcer) does not require anti-fungal treatment in the post-operative care of stable and immunocompetent patients. From a surgical point of view, at-risk groups are patients with severe post-operative (for instance suture insufficiency following esophagojejunostomy) or tertiary peritonitis [40], [41], [42], [43], [44], [45], [46]. Although the prophylactic administration of fluconazole has led to a reduction of Candida infections, it does not alter the lethality and is therefore not recommended [40], [47]. 

The number of Candida strains in Germany with limited sensitivity to fluconazole is approximately 40% [27], [40]. Therefore, against the backdrop of the results of recent multi-center studies, the use of an echinocandin (anidulafungin, caspofungin, micafungin) is preferred when the patient is unstable or when azole therapy or prophylaxis has recently been carried out. Alternatively, in cases of sensitivity to azoles and cardiovascular stability, the use of fluconazole and, if therapeutic drug monitoring is available, that of voriconazole may also be considered. Initial therapy with (liposomal) amphotericin B is also an option, taking into account the potential side effects (glomerular and tubular nephrotoxicity). There are no data available on isavuconazole or posaconazole in the treatment of invasive *Candida* infections (no grading). The duration of treatment is at least 14 days [40], [47]. Overall, there are few controlled data, especially for intra-abdominal mycoses. The published collectives are very heterogeneous as regards basic parameters [48]. Prognosis of IIM is poor in delayed treatment [40], [41], [42], [43], [44], [45], [46], [47], [48].

## Difficult to treat and multidrug- resistant pathogens (MDROs)

While in the mid-1990s 95–97% of all bacterial pathogens detected in IAI were still sensitive to common antibiotics (such as cefotaxime or ciprofloxacin + metronidazole), in recent years, especially in post-operative and tertiary peritonitis, the proportion of more resistant strains (MRSA, VRE, ESBL producer, (multi)resistant *Pseudomonas* spp.) have increased significantly worldwide [21], [22], [23], [27], [49], [50], [51], [52], [53]. It is important to cover the expected pathogen spectrum as completely as possible with the initial antibiotic treatment, particularly in the case of life-threatening clinical scenarios caused by resistant pathogens. If no evidence of resistant pathogens is found after microbiological examination, treatment should be de-escalated. 

An overview of the resistant pathogens and their frequency in intra-abdominal infections, which can also be used as a decision matrix for empirical treatment, is given in Table 6 (Tab. 6) in modified form [23]. The following sections deal with resistant pathogens, which require special considerations because of their particular importance. Information on the calculated treatment of IAI caused by these pathogens can be found in Table 2 (Tab. 3).

### MRSA

An infection of the abdominal cavity with MRSA is rare in immunocompetent patients. It is usually MRSA colonization due to an open abdomen, for example after abdominal compartment syndrome. In non-immunosuppressed patients an indication for antibiotic treatment results if local and systemic signs of infection and persistent evidence are present. In immunosuppressed patients after transplantation, any detection of MRSA should be considered as requiring treatment. Tigecycline is the only MRSA-active antibiotic approved for the treatment of IAI [54] and also covers the expected Gram-negative and anaerobic pathogen spectrum. Vancomycin is characterized by a relatively poor penetration into the abdominal compartment. There is clinical data on linezolid for the treatment of IAI [55], [56]. Linezolid, daptomycin and vancomycin should be combined with an antibiotic effective against Gram-negative pathogens [20], [21], [22], [23]. 

### Enterococci including VRE

The role of enterococci as the primary pathogen in a polymicrobial IAI is controversial [57], [58]. Enterococci of the species *Enterococcus faecalis* and *Enterococcus faecium* are important pathogens of nosocomial infections. They rank third in Germany in terms of frequency [27]. *Enterococcus faecium* has a broad spectrum of intrinsic and acquired antibiotic resistance and has become increasingly important as a pathogen for nosocomial infections in immunosuppressed and intensive care multi-morbid patients [20], [21], [22], [23], [27]. Enterococci-effective antibiotics should be used particularly in patients with postoperative peritonitis, tertiary peritonitis, severe abdominal sepsis and prior antibiotic treatment or endocarditis-prone patients (peritonitis and heart valve replacement), [18], [20], [21], [22], [23]. Compared to the rest of Europe, a comparatively high proportion of VRE*nterococcus faecium* is found in Germany [27]. Only a few antibiotics are active against VRE*nterococcus faecium*. These are tigecycline [59], linezolid [60], and also to some extent daptomycin (no controlled clinical data). Cases of linezolid or tigecycline-resistant enterococci strains have been documented, for instance [61].

### Resistant Enterobacteriaceae 

ESBL-producing Enterobacteriaceae can inactivate many of the penicillins and cephalosporins used for calculated initial treatment. In many cases there is also resistance to the beta-lactamase inhibitor-protected combinations amoxicillin/clavulanic acid, ampicillin/sulbactam, and (more rarely) piperacillin/tazobactam, as well as parallel resistance to other antibiotic classes, including fluoroquinolones and aminoglycosides [62], [63]. It can be assumed that there is now a huge reservoir of people worldwide who are colonized with ESBL-producing pathogens [27], [63] and that this is not a nosocomial phenomenon. To make matters worse, the proportion of ESBL-producing bacteria is also increasing significantly in animals and the pathogens are also detected on food and in water. In the PEG resistance study in 2013, the proportion of isolates with the extended spectrum beta-lactamase (ESBL) phenotype was 15.4% for *Escherichia coli* and 17.8% for *Klebsiella pneumoniae*. Furthermore, the proportion of carbapenemase-producing Enterobacteriaceae has also increased [27]. Further work on this important topic can be found in chapter 16 [64].

Carbapenems, fosfomycin (no monotherapy because of the risk of rapid development of resistance) and tigecycline are recommended as treatment of choice [18], [20], [21], [22], [23], [27]. The latter was used clinically in approximately 75% of patients with IAI under real-life conditions in a Europe-wide study [59]. Ceftolozan/tazobactam has recently become available as a new ESBL-effective drug. In the IAI licensing study, the ESBL-induced IAI group of patients was treated successfully under controlled conditions [25], [26]. Furthermore, the use of ceftazidime/avibactam, which was also recently approved for this type of indication, can also be considered [65].

By now, Enterobacteriaceae are also able to produce carbapenemases (KPC, NDM), thereby rendering carbapenem antibiotics ineffective. Occurrences of carbapenemases are most commonly found in *Klebsiella pneumoniae* (*Klebsiella pneumoniae* carbapenemases, KPC) but can also be found in *Escherichia coli* and other Gram-negative pathogens, for example *Acinetobacter* spp. Only a few options remain for adequate targeted treatment. Combination antibiotic regimens containing tigecycline, meropenem and colistin are recommended. The use of ceftazidime/avibactam, a new substance approved for this type of indication, can also be taken into consideration [66], [67], [68].

### Pseudomonas spp., Acinetobacter spp.

Pseudomonas spp. are detected in about 8–15% of all IAI, although the proportion of causally pathogenetically relevant strains is likely to be much lower [9], [20], [21], [69]. Resistance to 3 or 4 of the available antibiotic classes (3MRGN, 4MRGN) is also being observed more frequently in IAI. The same applies to carbapenem-resistant *Acinetobacter* spp., where in some cases tigecycline can still prove to be effective. As a special feature here occasionally sulbactam in monotherapy is also effective (note test result).

## Duration of empirical antibiotic treatment of intra-abdominal infections

Very often clinicians must begin antimicrobial treatment empirically, i.e. prior to pathogen detection. Recommendations for the empirical treatment of IAI according to a level model can be found in Table 4 (Tab. 4). The more local the infection is, the shorter the duration of treatment can be. For the treatment of community-acquired local peritonitis without risk factors (level 1: treatment duration 1 day; level 2: treatment duration 3 days), substances such as cephalosporins of groups 2 or 3a (for example cefuroxime, cefotaxime) or fluoroquinolones (for example ciprofloxacin), in each case in combination with metronidazole, should be used. Alternatively, aminopenicillins may be used in combination with a beta-lactamase inhibitor (for example amoxycillin/clavulanic acid) (see Table 3 (Tab. 3)) [18], [20], [21], [22], [23]. 

With increased local and systemic spread of the infection and possible risk factors (see Table 3 (Tab. 3)), piperacillin/tazobactam, ertapenem and, to a lesser extent, moxifloxacin may be considered (level 3: treatment duration 5 days). For advanced localized peritonitis, a 4–5 day treatment period is not inferior to an 8 to 10-day course of antibiotics with adequate infectious source rehabilitation, as recently demonstrated in a large randomized, double-blind study [70]. However, in nosocomial peritonitis and hemodynamically unstable patients in septic shock, the likelihood is high that resistant pathogens will co-trigger the infection. Only meropenem, imipenem, tigecycline (possibly in combination with a Pseudomonas-active substance) or ceftolozane/tazobactam + metronidazole or ceftazidime/avibactam + metronidazole should be used (level 4: treatment duration 7–10 days). If no treatment success occurs after 7–10 days, discontinuation of antimicrobial treatment and taking new samples is preferable to continuation of an unclear, resistant pathogen-selecting, potentially toxic therapy.

## Note

This is the seventh chapter of the guideline “Calculated initial parenteral treatment of bacterial infections in adults – update 2018” in the 2^nd^ updated version. The German guideline by the Paul-Ehrlich-Gesellschaft für Chemotherapie e.V. (PEG) has been translated to address an international audience.

## Competing interests

The authors declare that they have no competing interests.

## Figures and Tables

**Table 1:**
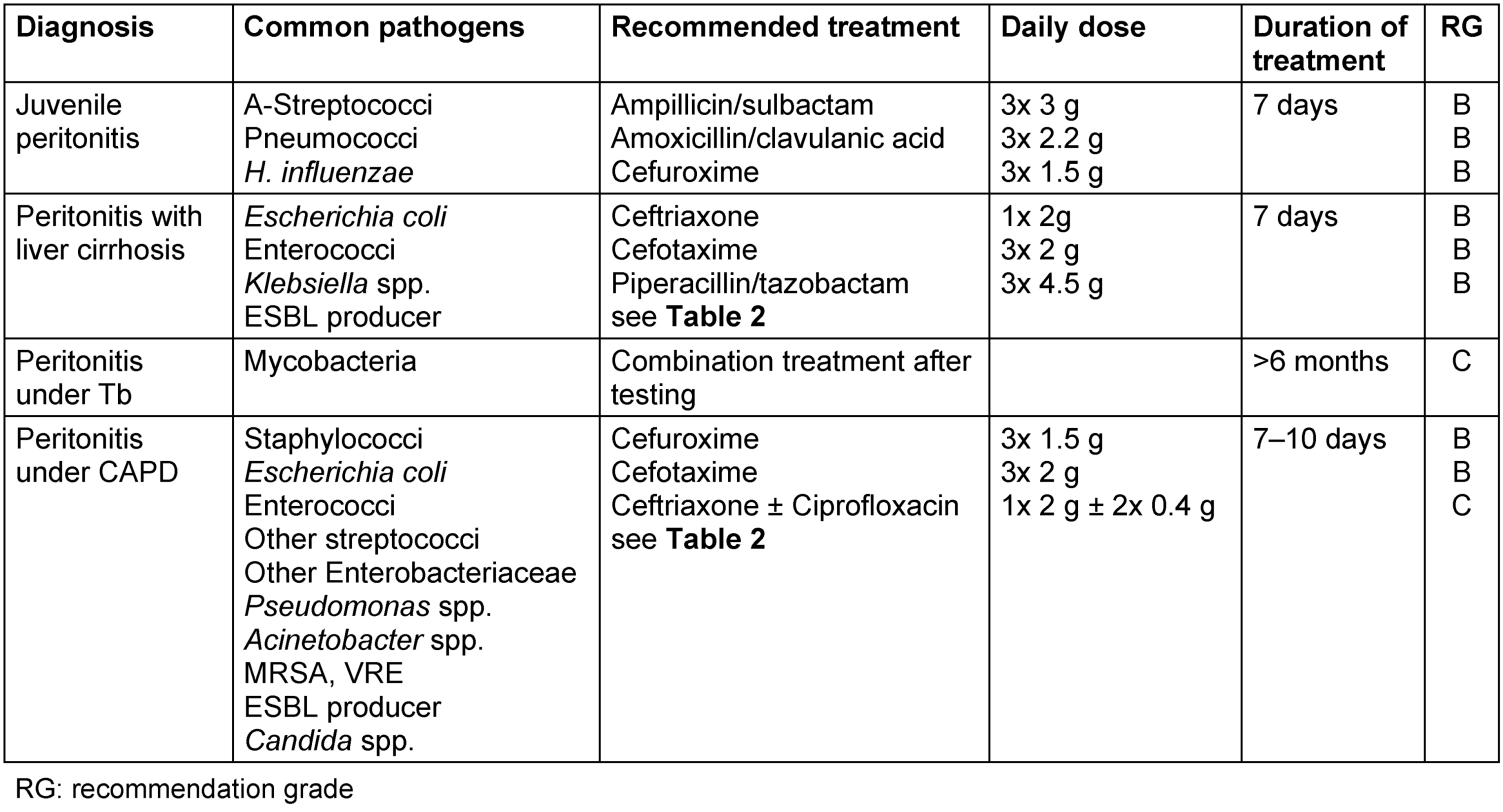
Therapy recommendations for the initial treatment of different forms of primary peritonitis

**Table 2:**
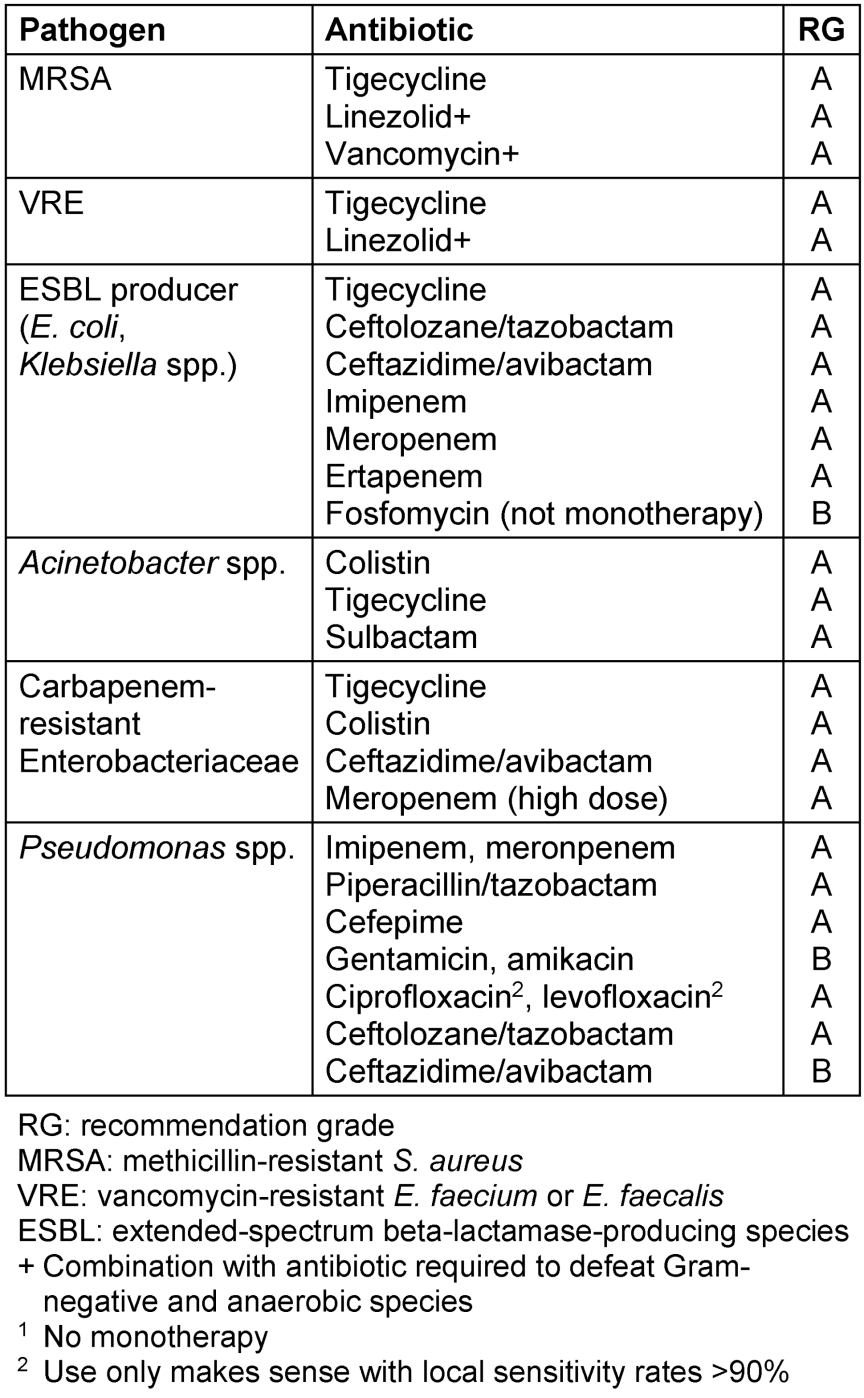
Calculated antibiotic treatment for intra-abdominal infection suspected of resistant pathogens

**Table 3:**
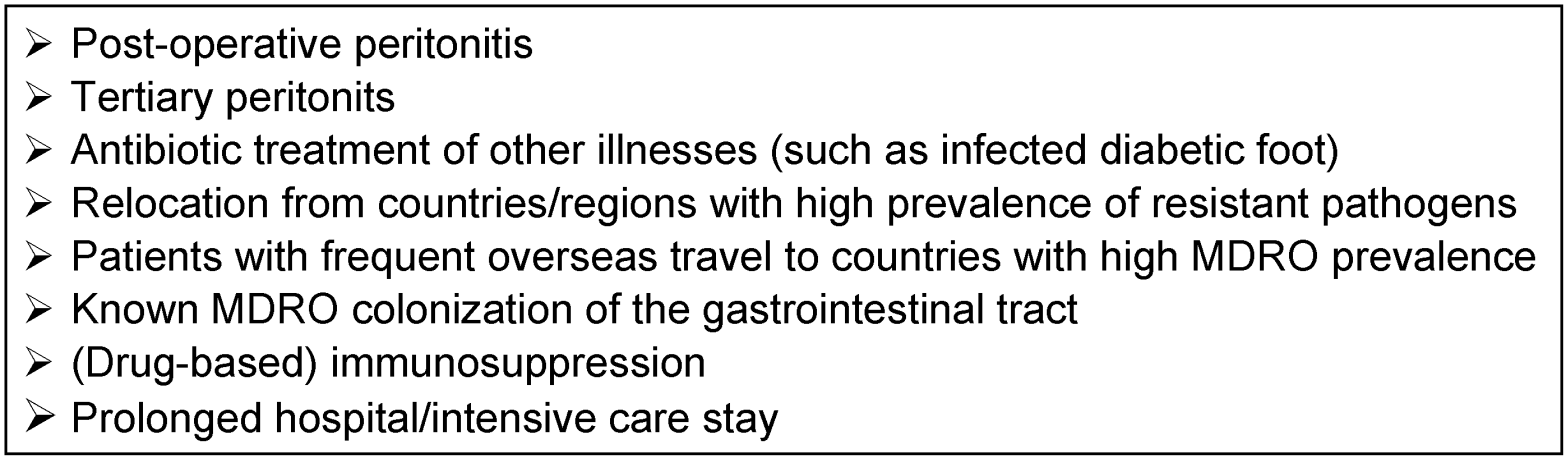
Evidence-based risk factors for the presence of multidrug-resistant pathogens (MDROs) in abdominal infections

**Table 4:**
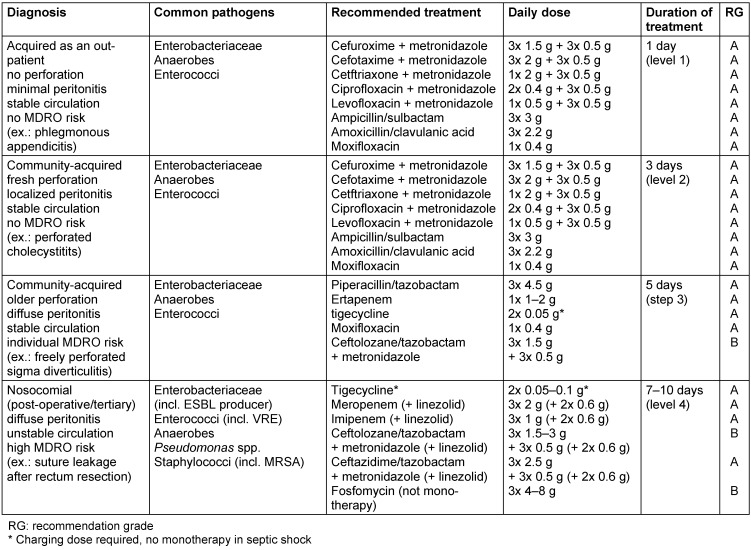
Recommendations for the initial treatment of the different forms of secondary and tertiary peritonitis

**Table 5:**
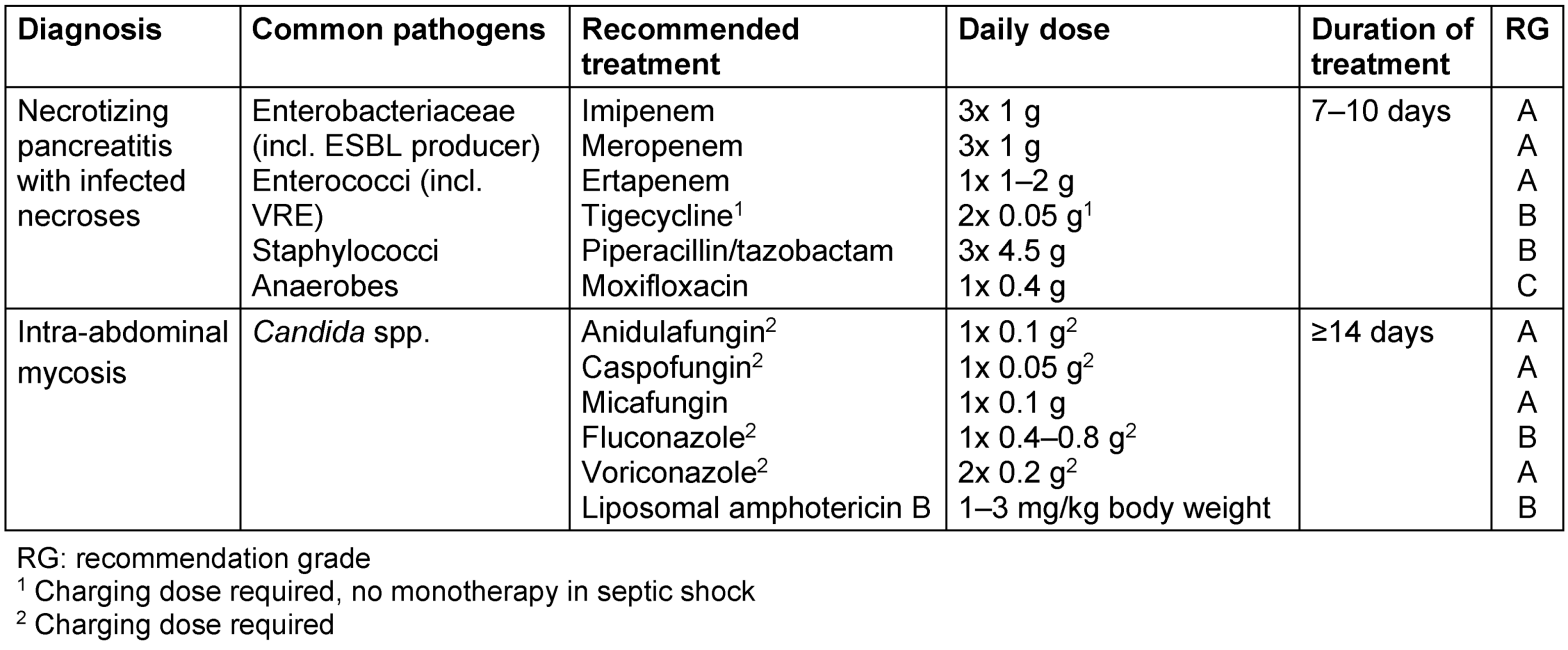
Calculated antibiotic therapy for necrotizing pancreatitis and intra-abdominal mycoses

**Table 6:**
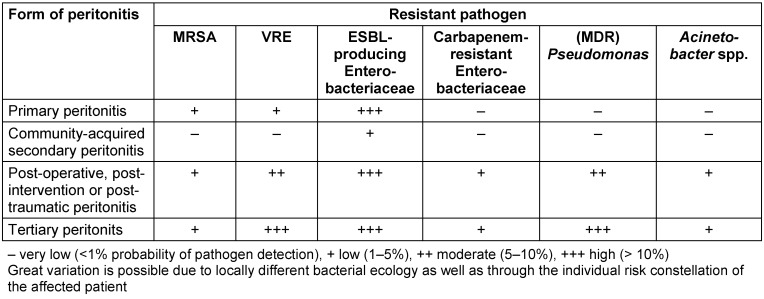
Matrix for the probability of detecting resistant pathogens in various forms of peritonitis (modified according to [23])
